# Cardiac CT and Transesophageal Echocardiogram Evaluation of a Sinus Venosus-Type Atrial Septal Defect With Partial Anomalous Pulmonary Venous Return and a Persistent Left Superior Vena Cava

**DOI:** 10.7759/cureus.20367

**Published:** 2021-12-12

**Authors:** Jenna M Mamatov, Jacob M Robinson, Edward Z Sanchez

**Affiliations:** 1 Osteopathic Medicine, Lake Erie College of Osteopathic Medicine, Bradenton, USA; 2 Radiology, Northside Hospital, Saint Petersburg, USA

**Keywords:** plsvc, svc, papvr, asd, persistent left superior vena cava, partial anomalous pulmonary venous return, atrial septal defect, sinus venosus

## Abstract

The sinus venosus (SV) plays a significant role in the embryological heart as the initial structure where the cardinal, umbilical, and vitelline veins drain before remodeling into the caval veins. As the human heart develops, the SV incorporates into the posterior wall of the right atrium. Sinus venosus atrial septal defects (SVASDs) result from a defect in the wall present among the right pulmonary veins, the superior vena cava (SVC), and the right atrium. Persistent left superior vena cava (PLSVC) occurs when the Marshall ligament does not regress, and in most cases, the PLSVC enters the coronary sinus before draining into the right atrium. Pulmonary hypertension from chronic left to right shunting makes recognizing this condition clinically significant. In this case report, both cardiac CT and transesophageal echocardiogram were used to further evaluate an SVASD with partial anomalous pulmonary venous return (PAPVR) of the right superior pulmonary vein, in addition to a PLSVC. The incidence of the co-occurrence of SVASD and PLSVC, as well as the association between the two, were discussed in this case report. Future research should focus on the potential genetic causes of this co-occurrence. It should also focus on patient treatment and outcomes at different stages of presentation to optimize patient management and improve mortality.

## Introduction

Atrial septal defects (ASDs) are communications between the atria walls that result in blood shunting from the left side of the heart to the right. Sinus venosus atrial septal defects (SVASDs) are the second rarest of the four types. The inherent nature of these defects commonly results in partial anomalous pulmonary venous return (PAPVR). Initially, SVASDs are asymptomatic, but long-standing ones can develop heart failure and poor patient outcomes [1]. Duplication of the superior vena cava (SVC), also known as persistent left superior vena cava (PLSVC), occurs in about three out of 1,000 people and is usually benign [2]. The co-occurrence of SVASD and PLSVC has occurred several times in literature. The prevalence in the population was not found, but it is thought to be rare [3-5]. Further analysis of such co-occurring cases could provide additional information regarding the clinical course, particularly whether or not a duplication increases the rate at which SVASD patients develop right heart failure.

## Case presentation

A 41-year-old Hispanic male patient presented to an outpatient heart failure clinic to establish care. His cardiac history was notable for a congenital ASD. Previously, this was followed and managed by a cardiologist outside of the United States. The patient reported that surgery was recommended to repair his ASD many years ago but was never completed. Past medical history was significant for congenital ASD with secondary chronic right ventricular combined heart failure. His severely reduced ejection fraction (EF) was secondary to his dilated non-ischemic cardiomyopathy [New York Heart Association (NYHA) functional classification III symptoms, American Heart Association (AHA)/American College of Cardiology (ACC) stage C]. Also, the patient recently had experienced an episode of atrial fibrillation resolved following electrical cardioversion. Valvular heart disease, including mild non-rheumatic mitral regurgitation and moderate-to-severe tricuspid regurgitation, were present. The cardiac history additionally noted moderate pulmonary hypertension, severe left ventricular hypokinesis, mild left ventricular hypertrophy, four-chamber heart dilatation, and an incomplete right bundle branch block (RBBB). Further medical history included small-to-moderate ascites, obesity, and erectile dysfunction attributed to beta-blocker usage. Past surgical history included ophthalmic procedures for bilateral congenital ocular defects. The patient denied any history of tobacco or alcohol use and reported that family history was significant for heart disease, hypertension, and diabetes mellitus. The patient had a hospital admission three months prior for the complaint of increased shortness of breath, abdominal distention, and swelling. At that time, an echocardiogram showed a severely reduced ejection fraction of 5-10%. Guideline-directed medical therapy was initiated, including spironolactone, losartan, furosemide, metoprolol succinate, and an external defibrillator device.

His vital signs revealed a blood pressure of 99/64 mm Hg, heart rate of 78 beats/minute (bpm), respiratory rate of 18 breaths/minute, O_2_ saturation (room air) of 98%, a weight of 88.7 kg, and a body mass index of 31.6 kg/m². He was a well-developed overweight male in no acute distress. His neck was supple and there was no jugular venous distention or carotid bruits noted. Cardiovascular exam demonstrated regular rate and rhythm, S1 and S2, with a soft 2/6 systolic murmur. No S3, S4, gallop, or rub was noted. Pulses were symmetric and 2+ in bilateral radial arteries, and capillary refill was reported as less than three seconds. No edema was recorded in the extremities. The respiratory pattern was even, non-labored, and clear, although slightly diminished breath sounds were identified posteriorly in the bilateral lung bases. The abdomen was obese, non-tender, and non-distended with active bowel sounds in all four quadrants. Table 1 lists the laboratory findings.

**Table 1:** Laboratory findings. NT: N-terminal, TSH: thyroid stimulating hormone, AST: aspartate aminotransferase, ALT: alanine aminotransferase, BUN: blood urea nitrogen.

Lab	Value	Reference range
White blood cells	9.18 k/µL	4.20-11.10
Segmented neutrophils	60.6%	40-69
Hemoglobin	18.6 g/dL	12.2-16.8
Platelets	187 k/µL	142-424
Sodium	136 mmol/L	136-145
Potassium	4.1 mmol/L	3.6-5.2
Glucose	128 mg/dL	70-110
NT-pro-B-type natriuretic peptide	1453 pg/mL	0-175
TSH	5.64 uIU/mL	0.36-3.74
Free T4	1.33 ng/dL	0.7-1.8
AST	65 units/L	15-37
ALT	129 units/L	30-65
Creatinine	1.30 mg/dL	0.55-1.30
BUN	27 mg/dL	7-18

**Figure 1:**
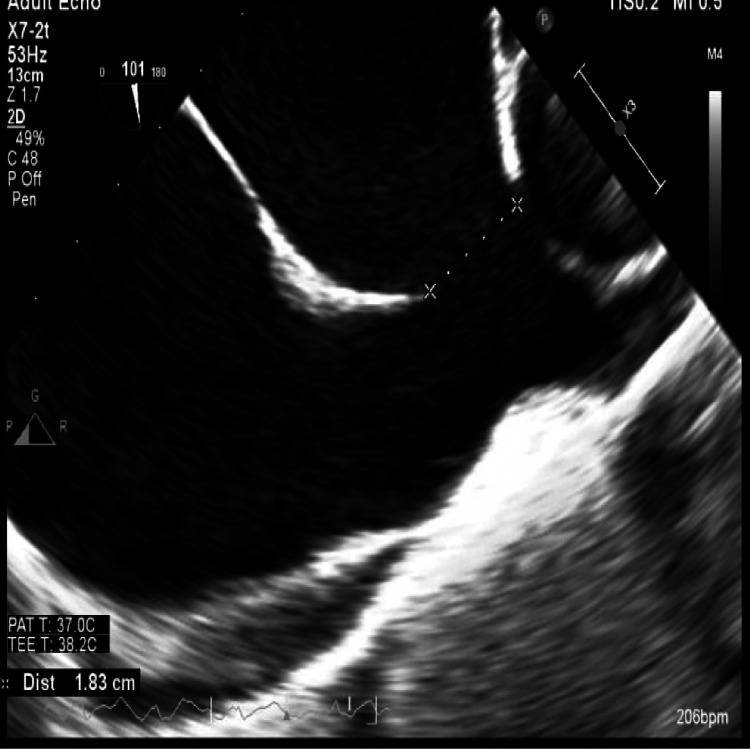
A grayscale image from a TEE shows a 1.83 cm superior SVASD. TEE: transesophageal echocardiogram, SVASD: sinus venosus atrial septal defect.

**Figure 2:**
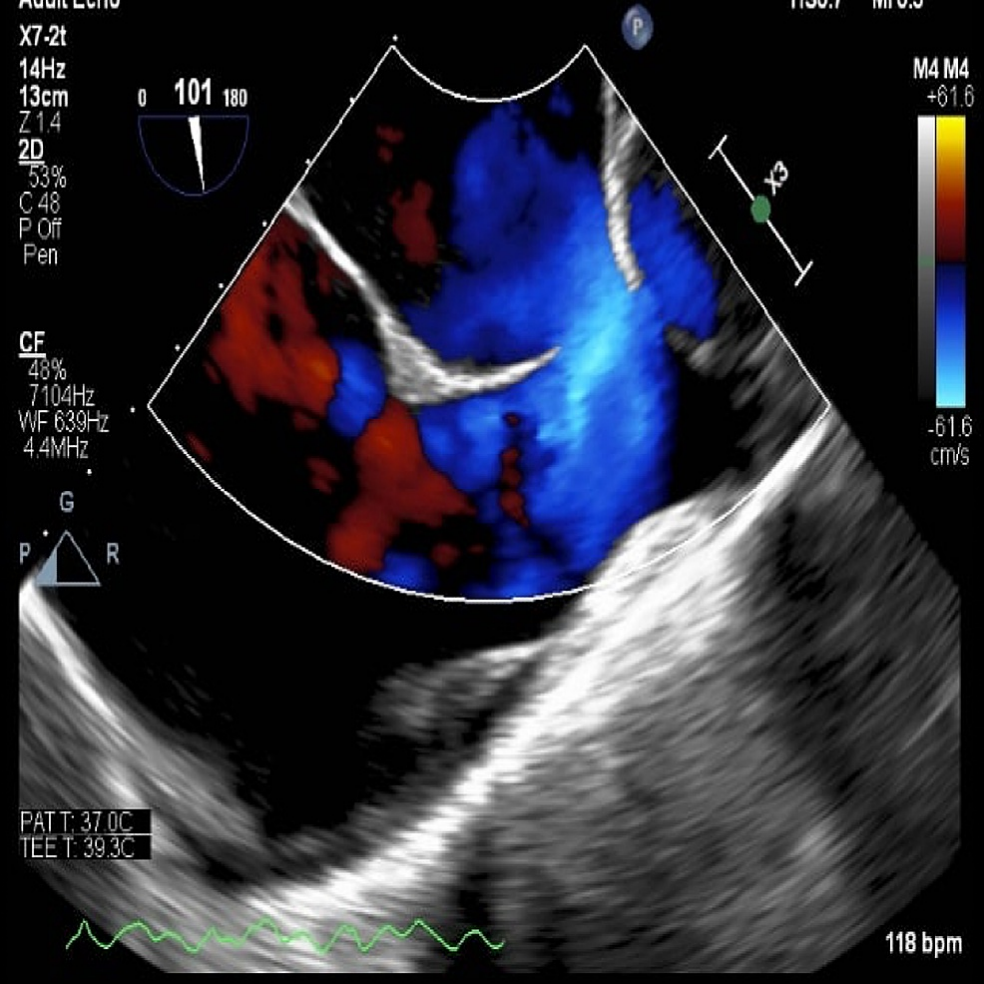
A color Doppler image from TEE shows a superior SVASD. TEE: transesophageal echocardiogram, SVASD: sinus venosus atrial septal defect.

A transesophageal echocardiogram (TEE) with electrical cardioversion was scheduled and performed on the same date as the cardiac CT. A repeat electrocardiogram (ECG) confirmed a normal sinus rhythm with a ventricular rate of 68 bpm. ECG also suggested possible left atrial enlargement, a rightward axis, and an incomplete right bundle branch block (RBBB). The TEE showed that the coronary sinus (CS) was dilated and the presence of a superior sinus venosus (SV) atrial septal defect measuring 1.9 × 1.8 cm (Figures 1, 2). The interatrial septum was observed to bow toward the left atrium suggesting increased pressure and volume in the right atrium. The left ventricular ejection fraction (EF) was estimated at 20-25% at this time with diffuse hypokinesis. There was trace tricuspid insufficiency and mild-moderate mitral insufficiency, which may have been underestimated due to the low EF. No left atrial appendage thrombus was appreciated.

**Figure 3:**
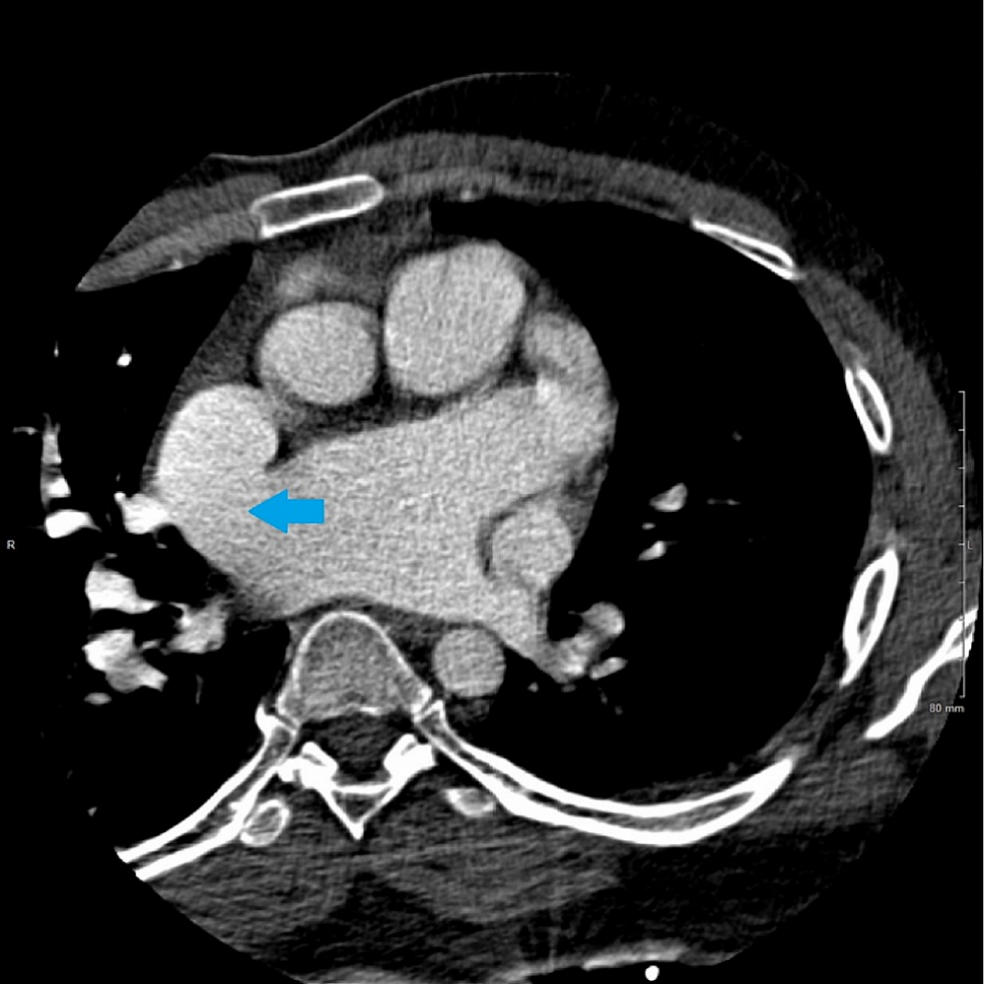
The intra-atrial septum is absent near the SVC compatible with an SVASD. The blue arrow is pointing to the SVASD. SVC: superior vena cava, SVASD: sinus venosus atrial septal defect.

**Figure 4:**
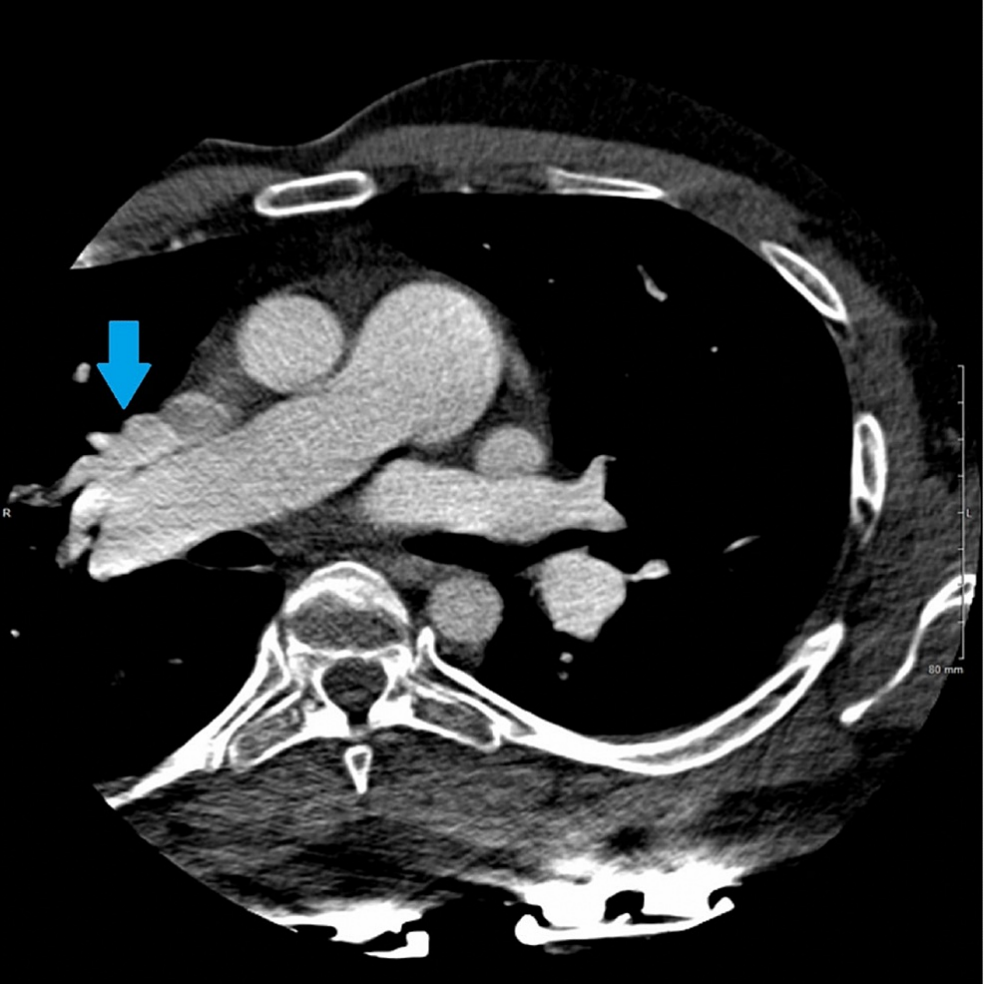
PAPVR of a right upper lobe pulmonary vein (blue arrow) is seen to drain into the right SVC instead of the left atrium. The main and right pulmonary arteries are dilated with respect to the ascending aorta. PAPVR: partial anomalous pulmonary venous return, SVC: superior vena cava.

**Figure 5:**
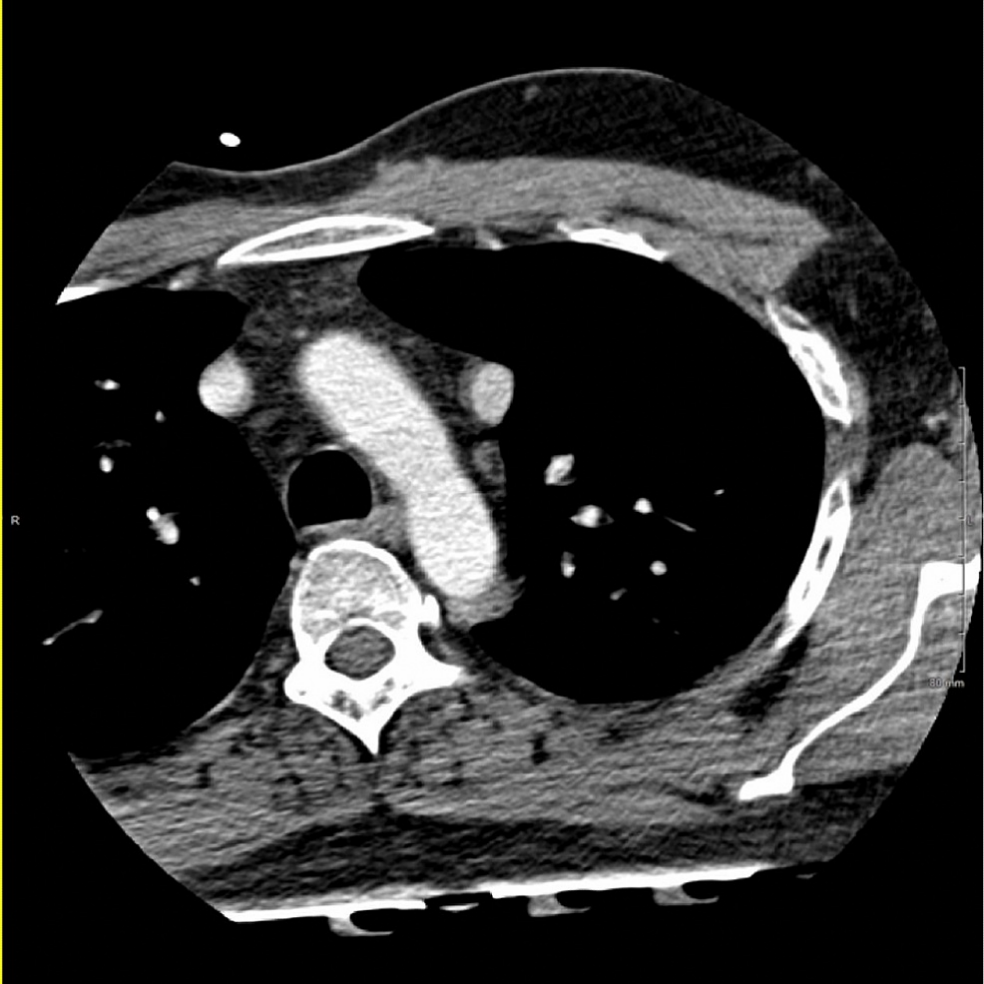
Axial CT image at the level of the aortic arch demonstrates a right SVC and a left SVC. SVC: superior vena cava.

**Figure 6:**
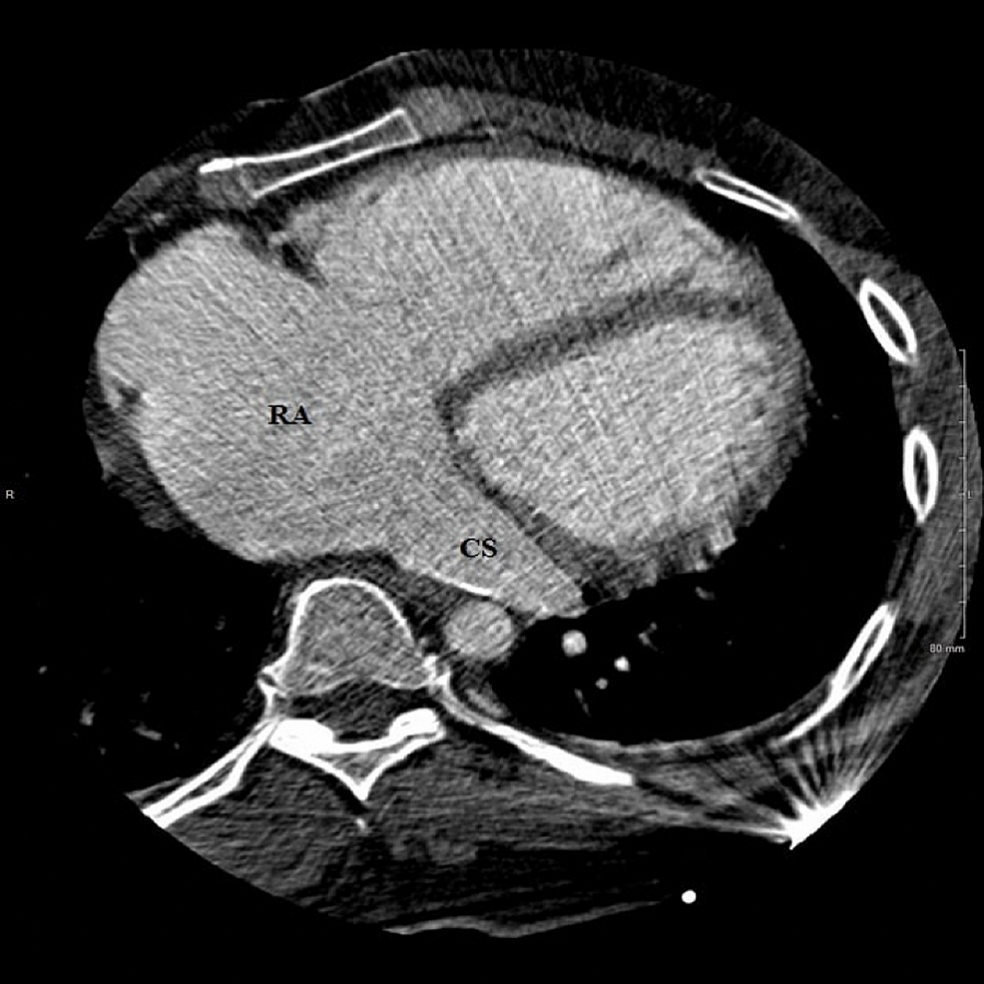
A CT image in the axial plane demonstrates multi-chamber cardiac enlargement. The PLSVC (not imaged) connects to a dilated coronary sinus which drains into the right atrium. PLSVC: persistent left superior vena cava, CS: coronary sinus, RA: right atrium.

Cardiac CT confirmed the presence of a large (1.9 × 2.1 cm) SVASD (Figure 3) with multi-chamber cardiac enlargement. A PAPVR of the right superior pulmonary vein was noted to drain directly into the right SVC (Figure 4). Upon further examination of the great vessels, the presence of a PLSVC was incidentally noted, which drained into a robust CS (Figures 5, 6). The main pulmonary and right pulmonary arteries were dilated with respect to the ascending aorta, suggesting a component of chronic pulmonary hypertension. This was further supported by the reflux of contrast medium from the right atrium into the inferior vena cava and hepatic veins. CT evaluation was limited to reformatted axial views as the images from the sagittal and coronal planes were obscured by overlying streak artifacts which resulted from the external defibrillator device.

Following his initial evaluation, the patient has continued to follow up in the outpatient heart failure clinic. A consult by a cardiothoracic surgeon specializing in adult congenital heart defects recommended surgical repair of the SVASD with oversewing of the left atrial appendage and a bi-atrial Cox-Maze procedure. At this time, the patient has not yet undergone this surgery and is awaiting scheduling.

## Discussion

Atrial septal defects are communications between the wall of the atria that result in blood shunting from the left side of the heart to the right. The four types are ostium primum, ostium secundum, coronary sinus ASDs, and SVASDs. Sinus venosus ASDs, present in our patient, are the second least common type, with the superior type comprising only 5-10% of all ASDs [1]. The SV plays a significant role in human heart development. It is the initial part of an embryological heart where the cardinal, umbilical, and vitelline veins drain before they remodel into the caval veins. As the human heart develops, the SV is eventually incorporated into the posterior wall of the right atrium [6]. The literature has described two types of SVASD: the superior vena caval type and the rarer inferior SVASD. The superior vena caval type, found in our patient, is a defect in the wall between the right pulmonary veins, the SVC, and right atrium resulting in partial anomalous pulmonary venous return. The rarer inferior SVASD results from a defect of the inferior or posterior part of the atrial septum, with one or both of the right pulmonary veins entering the right atrium anterior to the atrial septum [7,8]. In one article, the inferior subtype was only present in six of the 115 patients selected to participate in the study [8].

Diagnosis of an SVASD is usually made with diagnostic imaging. A normal physical exam does not preclude the presence of an SVASD, as most are initially asymptomatic. The typical auscultatory finding of someone with an ASD is a wide and fixed split of the S2 heart sound. An ECG can help guide a physician toward the diagnosis. An incomplete RBBB can present in all ASDs, and this finding was observed in our patient. In the inferior leads, inverted P waves can be seen in SVASDs, indicating a malfunctioning sinus node [1]. Inverted P waves were not present in our patient’s ECG. Chest radiographs can be abnormal in patients with long-standing SVASDs. Right heart dilation can be seen best in lateral films if present. The pulmonary arteries are usually also enlarged. Echocardiography can provide a definitive diagnosis of SVASD by determining the size, the shunt ratio, and pulmonary artery pressure estimations [1]; however, transesophageal echocardiography is usually required. Transthoracic echocardiography only detects about 12% of SVASDs. More advanced imaging, such as cardiac MRI or computed tomography angiography (CTA), may be required if the defect is not visualized on an echocardiogram [9].

Initially, most SVASDs are asymptomatic. However, long-standing ones can result in right heart failure due to chronic right atrial dilation and a left-to-right shunt, which had occurred in our patient. Signs and symptoms of right heart failure include dyspnea, fatigue, syncope, cyanosis, and edema. Exercise intolerance with dyspnea and fatigue is the most common initial presenting symptom [1,9]. An ASD generally has to be at least one centimeter in size to cause symptoms [1]. The SVASD in our patient measured approximately two centimeters in size. In 5-10% of patients with any ASD, pulmonary vascular disease develops [1]. In less than five percent of ASD cases, the continued hypertrophy of the pulmonary arteries and progressive pulmonary hypertension over time can result in Eisenmenger syndrome. Eisenmenger syndrome occurs when the blood flowing through the defect reverses direction. The left-to-right shunt, therefore, transforms into a right-to-left shunt. This occurs when the pulmonary pressure and pressure in the right side of the heart reach the systemic pressure and pressure in the left side of the heart [10]. The result is severe cyanosis, even at rest. The cardiologist suspected that this transformation might be beginning to occur in our patient.

Most types of ASDs have a chance of closing spontaneously in the days and months after birth if they are small [7]. This is generally untrue for SVASDs. Early correction via surgical closure is the best way to prevent complications in patients with long-standing large defects [1,9]. Our patient was recommended to have his corrected “10 years ago” by a cardiologist. For unknown reasons, this was not performed. The patient’s current clinical condition might have been prevented if the surgery had been completed much earlier. Indications for closure of ASD include right atrial or ventricular dilation on imaging, as well as an ASD with a minimum diameter greater than one centimeter on echocardiogram or Qp:Qs >1.5:1 on flow assessment using echocardiography or MRI [1]. After correction, the patient’s symptoms should resolve if the ASD has not resulted in significant atrial and ventricular dilation, valve problems, and pulmonary vascular disease [1]. Surgery should also not be performed in patients with pulmonary hypertension that is too far progressed or when severe left ventricular dysfunction is present. In these cases, the defect can act as a “pop-off” valve [1]. In the case of this patient, surgical correction was recommended.

Duplication of the SVC only occurs in about 0.3% of people but is present in 4.5% of patients with congenital heart disease [2]. It happens when the Marshall ligament does not regress [4]. In 82% of cases, patients have a typical right SVC and a PLSVC [2]. Like in our patient, most cases of a PLSVC enter the coronary sinus and then drain into the right atrium. Even though these patients are usually asymptomatic, hypotension, ischemia, cardiac arrest, and arrhythmia can result from coronary sinus irritation during central line or pacemaker placement. The performing physician must be aware that a duplicate SVC is present during cardiac procedures. Eight percent of PLSVC cases enter a pulmonary vein to drain into the left atrium, resulting in a right-to-left shunt and the threat of systemic emboli when performing procedures using catheters. Most PLSVCs do not require treatment because of their asymptomatic nature. Surgery is the treatment of choice, if required [2].

The prevalence of superior SVASDs and associated PAPVR co-occurring with a PLSVC is unknown. The co-occurrence of SVASD and PLSVC has been described several times in literature [3-5]. A patient presenting with symptoms of heart failure should receive a complete evaluation to exclude an undiagnosed SVASD. Future research should focus on the potential genetic causes of this co-occurrence. It should also focus on patient treatment and outcomes at different stages of presentation to optimize patient management and improve mortality.

## Conclusions

An SVASD is a rare occurrence in the general population. Duplications of the SVC are also a rare occurrence. However, the co-occurrence of these two conditions is extremely rare and has only been described a few times in literature. It is unknown if the presence of a PLSVC affects the long-term prognosis of someone with an SVASD or if the SVC duplication affects the rate at which right heart failure develops. Future research should focus on the role of genetics in the development of this disorder and evaluate for any potential associations with other medical conditions. Patient management also needs to be further researched at different stages of presentation. The early recognition of someone with this co-occurrence is vital due to poor patient outcomes without treatment.
